# An online RCT on behavioural expectations effects of COVID-19 certification policies in England

**DOI:** 10.1016/j.jvacx.2023.100389

**Published:** 2023-09-20

**Authors:** Freya Mills, Holly Carter, Liza Benny, Matt Barnard, Charles Symons

**Affiliations:** a Behavioural Science and Insights Unit, UK Health Security Agency, United Kingdom; b School of Psychology, University of Sussex, United Kingdom; c Evaluation and Social Research Unit, UK Health Security Agency, United Kingdom

**Keywords:** COVID-19 certification, Vaccination, Protective behaviours, Lateral Flow testing

## Abstract

**Objective:**

The aim of this study was to compare the effects of different types of COVID-19 certification policy on subsequent behavioural expectations.

**Design:**

4 × 2 between-subjects pre-registered randomised controlled trial.

**Method:**

In August 2022, participants (*n* = 2726) in England were presented with a scenario describing a rise in COVID-19 infections and the introduction of new protective measures. The protective measures described varied with regards to the setting (healthcare vs. recreational) and the type of policy (no certification vs. vaccination vs. vaccination *or* free Lateral Flow test vs. vaccination *or* Lateral Flow test at personal cost). Participants then answered questions on their expectations to receive another dose of the COVID-19 vaccine, to receive the seasonal influenza vaccine and to adhere to other protective behaviours following the announcement, as well as questions based on Self-Determination Theory, COVID-19 vaccine hesitancy and broader vaccine hesitancy.

**Results:**

We found no main effects of setting or type of certification on expectation to receive the next dose of the COVID-19 vaccine, to receive the seasonal influenza vaccine, or to adhere to other protective measures, when controlling for baseline expectations.

**Conclusions:**

These findings suggest that it is unlikely that the concept of certification, however it is framed, alters inclinations in the English population towards COVID-19 and seasonal flu vaccination or inclinations towards adhering to other protective behaviours within settings to which certification would apply. These findings are based on a hypothetical scenario and should be interpreted with caution.

## Highlights

• There were no significant differences in expectations to receive the next COVID-19 vaccine between certification conditions.
• There were no significant differences in expectations to receive the influenza vaccine between certification conditions.
• There were no significant differences in expectations to adhere to protective behaviours between certification conditions.
• These findings were present in both vaccine confident and vaccine hesitant participants.

## Introduction

**Fig. 1:**
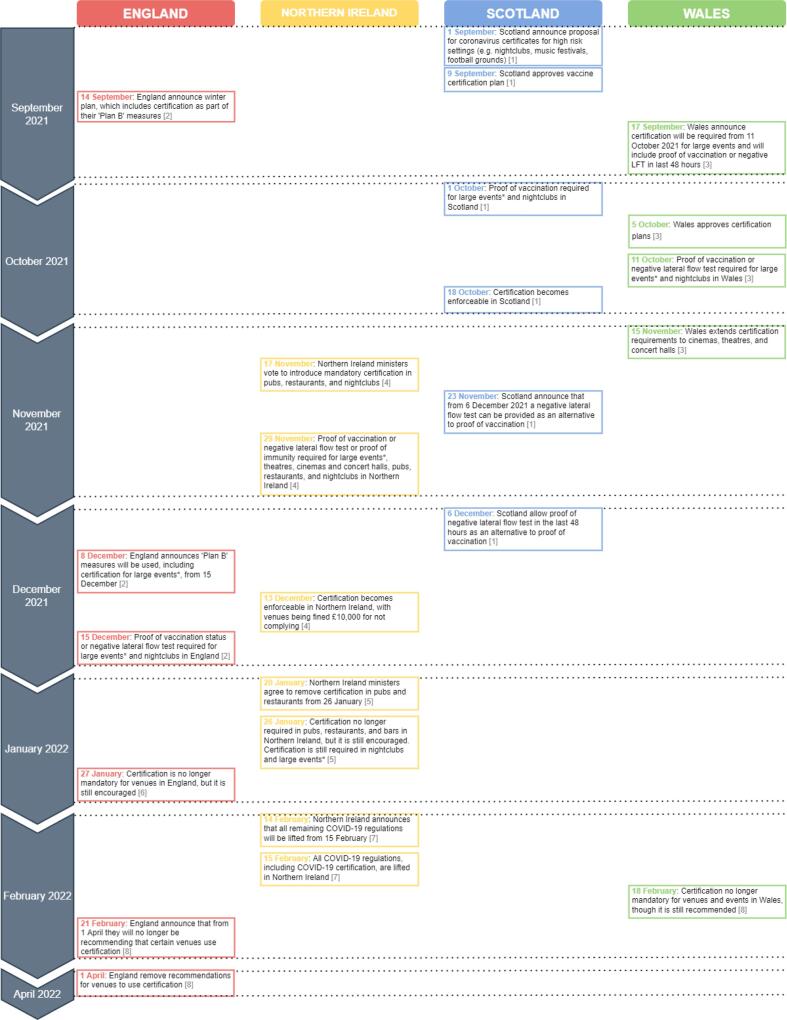
A timeline of key dates relating to COVID-19 certification in the UK.

COVID-19 certification refers to the presentation of an official document to suggest that an individual is at a lower risk from COVID-19. Certification schemes may include proof of vaccination status, current infectiousness, or current immunity. There was no universal certification strategy within the UK, with different certification schemes operating in England, Scotland, Wales and Northern Ireland. In December 2021, certification in England, Scotland and Wales required proof of vaccine status or a negative Lateral Flow Test (LFT), whereas Northern Ireland also included proof of natural immunity (Fig. 1 [1], [2], [3], [4], [5], [6], [7], [8]). The services and settings in which entry is dependent on certification can also differ, with certification potentially required for ability to work, or to access indoor hospitality, nightclubs, or large events [9].

There are several reasons that policymakers may implement certification, e.g., reducing transmission, hospitalisation and mortality risks in high-risk settings. However, it is important that policymakers consider both the ethics of enacting quasi-mandatory policies and whether intended health protection benefits are offset by other behavioural impacts such as reduced vaccination uptake amongst hesitant individuals [10], [11]. The focus of this particular study is on the health protection behaviour aspect.

### Impact of COVID-19 certification on COVID-19 vaccination

Previous research, using real-world data, suggests that both the announcement and implementation of certification may increase vaccine uptake [12], [13], [14], [15]. However, assessing the impact of certification on COVID-19 vaccine uptake is complex, as the extent to which certification has an effect depends on various factors including existing uptake [13], the setting in which certification is required [16], [17], [18], and existing attitudes towards COVID-19 vaccination [10]. Indeed, survey data from the UK and Switzerland showed greater acceptance of vaccine certification in high-risk settings, such as care homes, compared to social settings such as entertainment venues [16], [17], [18]. Furthermore, a UK cross-sectional survey indicated that vaccination intentions were lower amongst those uncertain about whether to receive the vaccine after the mention of vaccine certificates, particularly for domestic use (e.g., restaurants and nightclubs) [10]. This has been echoed in hypothetical experiments, whereby those with lower vaccination intentions elicit stronger psychological reactance when COVID-19 vaccine mandates are introduced [19], and in self-report data whereby unvaccinated individuals tend to argue more strongly against vaccine mandates [16], [20].

Such opposition may be explained by Self-Determination Theory (SDT), which posits that intrinsic motivation is based on individual needs for autonomy, relatedness and competence [21]. This has been explored in relation to vaccine certification, with one study finding that greater autonomy frustration (i.e., feeling forced to get the vaccine) was associated with reduced willingness to receive the COVID-19 vaccine, as well as predicting a decline in self-reported vaccination status, whereas relatedness satisfaction (i.e., believing that the authorities care and understand one’s needs) was related to an increase in both willingness to be vaccinated and in self-reported vaccine uptake [22]. These effects were greater when vaccine certificates were already in place and amongst those who were against vaccine passports. Similar concerns regarding autonomy restrictions have also been reported in interviews with the British population [23] and UK healthcare workers [24], [25]. One way in which such autonomy frustration could be reduced is by providing more options besides vaccination, such as negative LFTs, as this would enable individuals to choose their preferred method of signalling their COVID-19 status.

### Impact of COVID-19 certification on adherence to protective measures

Potential reductions in transmission and hospitalisation conferred by COVID-19 certification could be offset by a lack of adherence to protective measures within settings (e.g., social distancing, hand hygiene and wearing a face covering). There is a possibility that adherence may be attenuated due to an individual’s self-perceived reduction in susceptibility to COVID-19 [9]. While some research from previous disease outbreaks and before the COVID-19 vaccine rollout suggests that risky behaviours may increase after vaccination [26], [27], other research suggests no difference in adherence to protective behaviours amongst vaccinated and unvaccinated respondents, with overall adherence in fact increasing [28]. However, these studies do not consider the potential impact of certification on such behaviours.

### Impact of COVID-19 certification on seasonal influenza vaccine

It is also possible that COVID-19 certification could have a spill-over effect onto willingness to receive other vaccinations. An experimental study found that when the first dose of a hypothetical vaccine was compulsory, there was a 39% reduction in uptake of the second vaccine due to increased anger towards the mandate, particularly for those with existing negative attitudes towards vaccine uptake [29]. A similar hypothetical study exploring a mandatory COVID-19 vaccine policy found that subsequent intentions to receive the influenza vaccine decreased when participants with a negative attitude towards mandates were told to be forced to vaccinate against COVID-19 [30]. The present study explores this further.

### The current study

Thus far, the literature has explored the impact of the announcement and introduction of certification on uptake of the COVID-19 vaccine, as well as considering potential mitigating factors (e.g., existing uptake). However, there is a lacuna regarding how nuances in certification may affect subsequent behavioural outcomes, particularly amongst those hesitant towards the vaccine. Aspects that warrant further investigation include the impact of certification requirements in healthcare and recreational settings and the impact of including Lateral Flow testing (either free or at cost). By providing more options within certification, it is hypothesised that autonomy frustration could be reduced, and behavioural impact mitigated. This study aimed to address the following research questions:

1. Does the type of hypothetical COVID-19 certification policy affect expectations regarding subsequent behavioural outcomes?
2. Does the type of setting to which COVID-19 certification policy is applied affect expectations regarding subsequent behavioural outcomes?
3. Is there an interaction effect between type of hypothetical COVID-19 certification policy and setting to which COVID-19 certification policy is applied on expectations regarding subsequent behavioural outcomes?
4. Does baseline COVID-19 vaccination status moderate the relationship between certification policy parameters and expectations regarding subsequent behavioural outcomes?

All hypotheses and secondary research questions can be found in the pre-registered protocol. We hypothesised that, while controlling for baseline expectations, all types of COVID-19 certification would reduce subsequent behavioural expectations to receive the COVID-19 vaccine compared to conditions in which no certification is required, with expectations being lower when testing is not provided as an alternative to vaccination. We also hypothesised that the vaccination plus LFT at cost condition would result in lower expectations to receive the COVID-19 vaccine than when LFTs are included at no cost. Finally, we hypothesised that this effect would be moderated by baseline vaccination status, with effects being present in the vaccine hesitant cohort and not the vaccine confident cohort.

## Method

The reporting of the study follows the Consolidated Standards of Reporting Trials (CONSORT; Schulz et al. [31]) reporting guideline (Supplementary File 1). Ethical approval was obtained from UKHSA’s Research Ethics and Governance Group (R&D 501). All participants gave informed consent before participating. The study analysis plan was pre-registered, with the full protocol, on 16th August 2022 at: https://osf.io/8dhn6 [32].

### Design

This study was an online RCT with a 4 × 2 between-subjects design. Participants were randomly assigned to one of eight groups, varying by setting (healthcare vs. recreational) and policy (no certification vs. vaccination-only vs. vaccination status *or* free negative LFT vs. vaccination status *or* negative LFT at cost; Table 1).

**Table 1:** Intervention design.

	No certification	Vaccination	Vaccination plus free testing	Vaccination plus testing at cost
Healthcare	Introduction of face coverings and physical distancing in care homes and hospitals	Introduction of face coverings, physical distancing and certification in care homes and hospitals. Certification requires receipt of at least three doses of the COVID-19 vaccine.	Introduction of face coverings, physical distancing and certification in care homes and hospitals. Certification requires receipt of at least three doses of the COVID-19 vaccine or demonstration of a free negative Lateral Flow Test in the last 48 hours.	Introduction of face coverings, physical distancing and certification in care homes and hospitals. Certification requires receipt of at least three doses of the COVID-19 vaccine or demonstration of a negative Lateral Flow Test in the last 48 hours (tests need to be purchased).
Recreational	Introduction of face coverings and physical distancing in nightclubs and large indoor and outdoor settings	Introduction of face coverings, physical distancing and certification in nightclubs and large indoor and outdoor settings. Certification requires receipt of at least three doses of the COVID-19 vaccine.	Introduction of face coverings, physical distancing and certification in nightclubs and large indoor and outdoor settings. Certification requires receipt of at least three doses of the COVID-19 vaccine or demonstration of a free negative Lateral Flow Test in the last 48 hours.	Introduction of face coverings, physical distancing and certification in nightclubs and large indoor and outdoor settings. Certification requires receipt of at least three doses of the COVID-19 vaccine or demonstration of a negative Lateral Flow Test in the last 48 hours (tests would need to be purchased).

Previous research suggests that the impact of vaccine certification varies according to existing vaccination perceptions and behaviour [10]. Thus, this design ran two concurrent experiments with two cohorts: 1) ‘Vaccine Confident’ (having received or booked at least three doses of the COVID-19 vaccine) and 2) ‘Vaccine Hesitant’ (having received or booked fewer than three doses of the COVID-19 vaccine). To ensure an equal number of participants in each cohort, a pre-screening survey was conducted to identify the number of doses participants had received, and therefore which participants would be allocated to each cohort.

### Participants

Full details of the power calculation can be found on the pre-registered protocol. A power calculation using G* Power 3.1.9.2 indicated that the study required 2381 participants for an ANCOVA, achieving 95 % and small effect size (*f* = 0.085) [33]. To account for participants failing both attention check questions (10%) and for a potential attrition (15%), we aimed to recruit 3012 participants for the pre-screening study.

Participants were recruited from a representative sample of the UK using the recruitment website Prolific. Participants were invited to take part if they met the following inclusion criteria: i) being aged 18 or above; ii) living in England since the beginning of 2021; iii) fluent in written English; iv) had not had an application approved for medical exemption from vaccination and/or testing for COVID Pass (aside from temporary exemption). The sample was based in England to reduce potential confounds that may occur due to personal past experience with different COVID-19 certification schemes across the UK.

We recruited 3020 participants in the pre-screening study and 2771 in the final study. 45 participants were removed from the final study due to incomplete measures, leaving a final sample size of 2726 (Fig. 3). All participants correctly responded to at least one attention check. Participants were reimbursed through Prolific with a monetary compensation of approximately £2.00. The survey was piloted with 66 participants to check randomisation procedures and question coherence and to obtain any feedback. No changes were required following the pilot.

### Materials

**Fig. 2:**
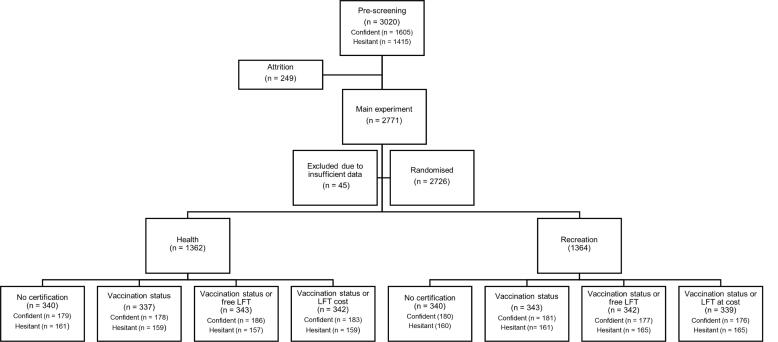
Flow diagram of participant allocation to scenario treatment arms.

Participants were presented with a hypothetical scenario, developed by the authors. Scenarios began by describing an increase in COVID-19 infections in England and requirement of new protective measures, followed by one of eight descriptions of hypothetical countermeasures varying with regards to the type of setting requiring such countermeasures and the type of countermeasures in place (Fig. 2). The control condition described a requirement for face coverings and social distancing, unless exempt, and explained that more information could be found on gov.uk. The intervention scenarios included the same information, in addition to requiring the NHS COVID Pass with either proof of having received three doses of the COVID-19 vaccine or proof of a negative LFT (either free or at a personal cost). See Supplementary File 2 for a copy of the scenarios and their word count and predicted readability.

### Measures

The questionnaire can be found in Supplementary File 3. Unless otherwise stated, all response options used a 5-point Likert scale from 1 (e.g., very unlikely) to 5 (e.g., very likely).

#### Primary outcome measures

Participants’ expectations to receive their next COVID-19 vaccine (baseline: α = 0.96; outcome: α = 0.97) was assessed using four questions taken from Gursoy et al [34]. Participants’ expectations to receive other types of vaccination (baseline: α = 0.94; outcome: α = 0.95) was measured using the same questions and response options used for measuring COVID-19 vaccination but with “COVID-19” replaced with “seasonal flu” throughout.

Three questions exploring participants’ expectations to adhere to protective behaviours (baseline: α = 0.80; outcome: α = 0.88) were created by the study authors. These questions asked how likely or unlikely participants would be to wear a mask, apply hand sanitiser and maintain physical distancing following the announcement.

#### Covariate measures

Primary outcome measures were measured both before and after presentation of the scenario to allow for controlling of baseline expectations. Additional covariates were measured to test for effects of conditions on seasonal flu vaccination. These included: receipt of the seasonal flu vaccine last winter; recommended to receive the flu vaccine; eligibility for a free flu vaccine.

#### Moderation measures

The baseline COVID-19 vaccination status measure was adapted from de Figueiredo et al [10]. These questions asked participants to choose an option that described their current situation, with scales being adapted to include the booster doses of the COVID-19 vaccine and to include options for having booked an appointment to receive a vaccine. Moderators for secondary research questions included the Vaccine Concerns in COVID-19 Scale (VaCCs; [35]) and Adult Vaccine Hesitancy Scale (AVHS; [36]).

#### Mediation measures

Mediation measures based on SDT constructs were adapted from Porat et al [22]. Each construct (autonomy, competence, and relatedness) contained a satisfaction scale (where the psychological need is met) and a frustration scale (where the psychological need is not met). These measures assessed participants’ autonomy (overall α = 0.84): satisfaction (α = 0.84) and frustration (α = 0.81); competence (overall α = 0.86): satisfaction (α = 0.88) and frustration (α = 0.91); and relatedness (overall α = 0.87): satisfaction (α = 0.87); and frustration (α = 0.86). The measures were adapted to be in the conditional tense about a future COVID-19 vaccine and to include other behaviours. The items on other behaviours were added to reduce demand awareness and were therefore not included in any analyses. For the exploratory analyses, the satisfaction and frustration scores for COVID-19 vaccination were summed to create single scores for each construct; frustration scores were re-coded so that a higher overall score meant lower autonomy, competence, or relatedness.

#### Additional measures

Two visualisation questions (α = 0.67; Carter et al. [37]) were asked and three manipulation checks. For policy, participants were presented with a list of potential COVID-19 countermeasures and asked to select all that were mentioned. For setting, participants were asked to select which settings were mentioned in the scenario. Individuals in the testing conditions were also asked to estimate how much the LFTs would personally cost.

In line with Prolific’s guidance [38] two attention checks were asked. Participants were also asked various demographic questions.

### Procedure

**Fig. 3:**
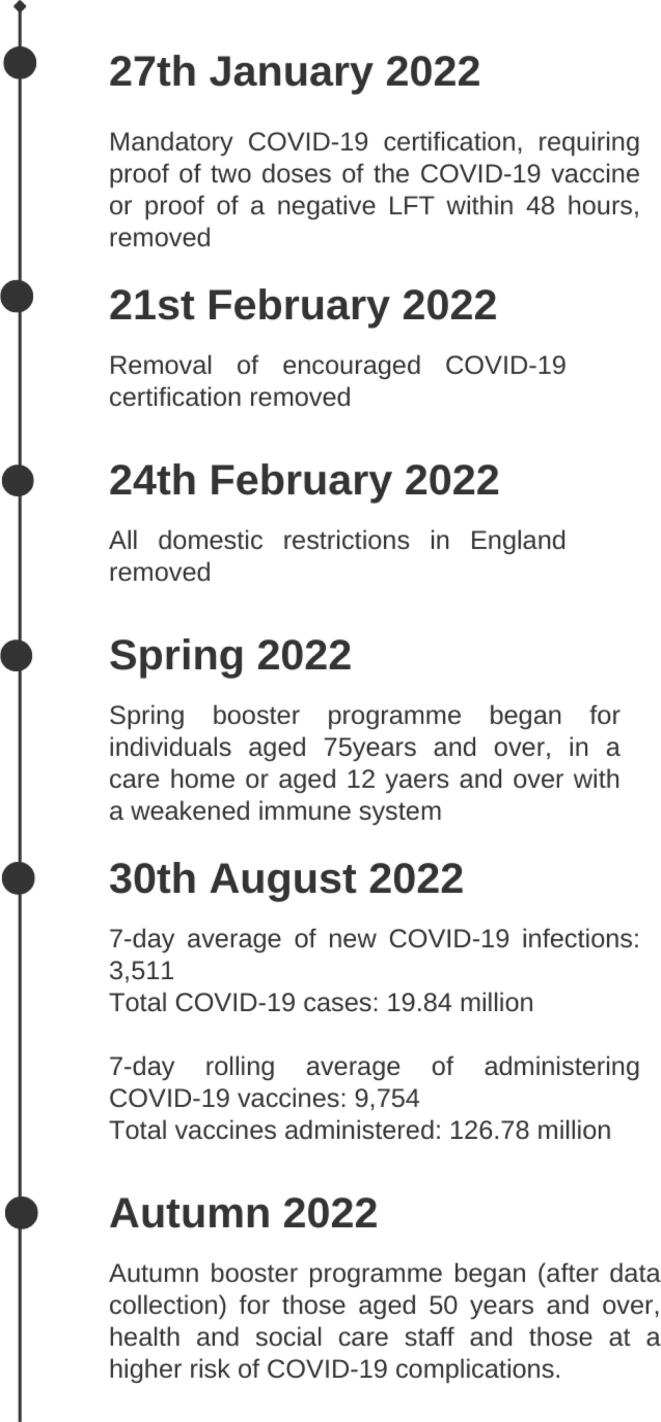
Timeline of key information regarding COVID-19 restrictions, infections and vaccinations at the time of recruitment.

The data were collected in England between 19th and 30th August 2022. At this time, there were no COVID-19 restrictions in place in the UK (Fig. 3, [39], [40], [41]) and the UK were offering the Spring Booster vaccine to some of the population (Table 2).

**Table 2:** Number of COVID-19 vaccines doses administered in England.

**Vaccine dose**	**7-day rolling average on 30th August 2022**	**Total administered by 30th August 2022**	**Percentage of the population aged 12 and over who had received the dose by 30th August 2022**
First	1659.30	45,233,342	93.5 %
Second	3927.10	42,629,512	88.1 %
Booster or third	2923.30	33,514,047	69.3 %
Spring booster	414.86	4,212,214	6.3 %

Note: data gathered from the COVID-19 dashboard [42]. The spring booster was not offered to the whole population.

The study was two-fold: a pre-screening survey and the RCT. The pre-screening survey consisted of 1-item asking participants how many doses of the COVID-19 vaccine they had received and/or booked. Based on these responses, participants were allocated to either the 1) ‘Vaccine Confident’ cohort or 2) ‘Vaccine Hesitant’ cohort. Two identical RCTs were then conducted in Prolific using Prolific’s custom allowlist to allocate participants to their respective experiment. The two RCTs contained the same questions and scenarios.

All participants were presented with the same questions. Participants were first presented with the baseline questions, assessing existing COVID-19 and influenza vaccination behaviour, and baseline versions of the post-intervention behavioural expectations measures. Participants were then randomly assigned to receive one of eight hypothetical scenarios. Participants were randomly assigned through Qualtrics, which means that researchers were blinded to the randomisation until the analysis. When downloading data, one researcher (CS) applied an anonymous code to each scenario so that the analyst (FM) was unaware of participants’ allocation during analysis.

Next, participants were presented with visualisation checks and their first attention check, followed by the primary outcome measures, SDT measures, manipulation checks, VaCCS and AVHS. Participants were then asked a final attention check and the demographic questions.

### Data analysis

Data analysis was carried out in IBM SPSS v27. The analyst was partially blinded to the conditions to which participants had been allocated. Full blinding, screening and coding procedures can be found in Supplementary File 4.

#### Preliminary checks

Descriptive statistics were used to describe demographics. Chi-square tests were used to conduct the manipulation checks. Two-way ANOVAs were applied to check whether there were differences between conditions in terms of perceived visualisation with the scenario.

#### Primary analyses

The internal consistency of scale items was measured using Cronbach’s Alpha. Three two-way ANCOVAs, with Bonferroni-corrected comparisons, were used to assess the effect of type and setting of certification on each of the three main outcome measures [43]. Bootstrapping (1,000 samples) was applied due to violation of assumptions of normality, indicated by Kolmogorov–Smirnov and Shapiro–Wilk statistics, for all behavioural variables.

To assess the moderating role of baseline vaccination status a moderation analysis was conducted using Model 1 of the PROCESS macro for SPSS [44]. The indicator coding system was used for any multicategorical variables.

#### False discovery assessment

Given the high number of statistical tests carried out, the Benjamini–Hochberg procedure [45] was applied with the false discovery rate (Q) set at 0.1 (Supplementary File 5). The *p* values used for interpretation from any extra, unregistered exploratory analyses were also included.

#### Sensitivity checks

The primary analyses were repeated with 31 cases removed that met the following criteria: submitted an incorrect response to one of the two attention checks; were incorrect or partially incorrect on one or more manipulation checks; and did not contact the research team to notify of an incorrect attention check.

## Results

### Demographics

Participants (*N =* 2726) aged 18 to 87 (*M =* 43, *SD* = 15.19) completed the study. The sample primarily consisted of people in full time employment (45.1%) and 51.3% of the sample either had or were working towards an undergraduate or postgraduate degree. 52.8% of the sample were categorised as Vaccine Confident (had received or booked at least three doses of the COVID-19 vaccine) and 47.2% of the sample were categorised as Vaccine Hesitant (had received or booked fewer than three doses of the COVID-19 vaccine). Sample characteristics can be found in Table 3. There were no significant differences in demographics or baseline expectations between the setting and policy conditions suggesting that randomisation was successful. There were significant differences in age, *t* (2711) = 17.16, *p* <.001, ethnicity, χ2(17) = 55.07, *p* <.001, and education, χ2(8) = 37.21, *p* <.001, between the vaccine confident and vaccine hesitant cohorts, with the vaccine confident cohort having a higher mean age; a higher count of participants of British ethnicity; and a higher count of participants with a postgraduate degree or higher as their highest educational qualification achieved to date. The hesitant cohort had higher counts of participants of Caribbean, Pakistani, Bangladeshi, African, and Mixed White and Black Caribbean ethnicities and a higher count of participants with A-Level or equivalent as their highest educational qualification achieved to date than the vaccine confident cohort. There were also significant differences in baseline expectation to receive the next dose of the COVID-19 vaccination, *t* (2724) = 38.73, *p* <.001, to get the flu vaccination, *t* (2724) = 30.70, *p* <.001 and to adhere to protective measures, *t* (2724) = 11.41, *p* <.001, between the vaccine confident and vaccine hesitant cohort with all expectations being higher in the vaccine confident condition.

**Table 3:** Participant Demographics.

**Participant Characteristic**		**N**	**%**
**Gender**			
	Female	1400	51.4
	Male	1309	48.0
	Non-binary or genderqueer	7	0.3
	Prefer not to say	8	0.3
**Sex**			
	Female	1411	51.8
	Male	1307	47.9
	Prefer not to say	6	0.2
**Ethnicity**			
	Asian / Asian British	186	6.3
	Black / African / Caribbean / Black British	79	2.9
	Mixed / Multiple Ethnic Groups	46	1.7
	White	2376	87.1
	Other	12	4.4
	Prefer not to say	20	7.3
**Education**			
	No qualification	30	1.1
	GCSE or equivalent	447	16.4
	A Level or equivalent	544	20.0
	Undergraduate degree	967	35.5
	Postgraduate degree or higher	430	15.8
	Professional qualification	208	7.6
	Trade apprenticeship	43	35.5
	Other	21	0.8
	Prefer not to say	14	0.5
**Region of England**			
	East Anglia	225	8.3
	East Midlands	243	8.9
	London	333	12.2
	North East	155	5.7
	North West	384	14.1
	South East	505	18.5
	South West	277	10.2
	West Midlands	287	10.5
	Yorkshire & Humber	296	10.9
	Prefer not to say	12	0.4
**Employment**			
	Full time employed	1233	45.2
	Part time employed	401	14.7
	In paid work with a zero-hours contract	16	0.6
	Self-employed	257	9.4
	Full time education	119	4.4
	Not in paid work	290	10.6
	Retired	298	10.8
	Other	66	2.4
	Prefer not to say	12	1.1
**Income**			
	Under £6,500	69	2.5
	£6,501 - £15,000	292	10.7
	£15,001 - £30,000	684	25.1
	£30,001 - £40,000	425	15.6
	£40,001 - £50,000	319	11.7
	£50,001 - £65,000	351	12.9
	£65,001 - £95,000	258	9.5
	£95,001 and over	141	5.2
	Don’t know	22	0.8
	Prefer not to say	162	5.9
**Vaccination status**			
	Has not received the COVID-19 vaccine	394	14.4
	Has had one dose of the COVID-19 vaccine	81	2.9
	Has had two doses of the COVID-19 vaccine and has not booked an appointment to have the third	811	29.8
	Has had two doses of the COVID-19 vaccine and has booked an appointment to have the third	18	0.7
	Has had three doses of the COVID-19 vaccine and has not booked an appointment to have the fourth	1319	48.4
	Has had three doses of the COVID-19 vaccine and has booked an appointment to have the fourth	21	0.8
	Has had four doses of the COVID-19 vaccine	82	3.0
**Vaccine Confident or Hesitant**			
	Confident	1440	52.8
	Hesitant	1286	47.2

Note: demographic subgroups are clustered to safeguard anonymity in cases where n per subgroup is less than 10.

### False discovery assessment and sensitivity check

The largest *p* value that was equal to or less than its Benjamini–Hochberg critical value was < 0.001. The interpretation of the analyses did not change when carrying out sensitivity checks.

### Manipulation and visualisation checks and exploratory analyses

Table 4, Table 5 present the findings from the manipulation checks. We conducted a chi-squared test to examine the association between the type of policy and the countermeasures reported by participants. We found that more people reported vaccination as a countermeasure in all three conditions requiring vaccination, compared to the no-certification condition, χ2(3) = 1181.08, *p* <.001. We also found that more people reported requiring an LFT in the two conditions mentioning LFTs than the vaccination-only and no-certification conditions, χ2(3) = 1886.16, *p* <.001.

**Table 4:** Percentage of participants in each policy condition reporting manipulation check responses pertaining to type of certification policy.

**Manipulation check**	**Policy condition**			
	No-certification (%)	Vaccination-only (%)	Vaccination plus free LFT (%)	Vaccination plus LFT at cost (%)
Reporting vaccination as a countermeasure	22.1	91.5	88.3	89.3
Reporting negative LFT as a countermeasure	6.3	7.4	90.8	89.1
Reporting LFTs as free	N/A	N/A	67.6	31.6

**Table 5:** Percentage of participants in each setting condition reporting manipulation check responses pertaining to setting requiring certification.

**Manipulation check**	**Setting condition**	
	Healthcare (%)	Recreational (%)
Reporting care homes as a setting requiring countermeasures	94.9	3.0
Reporting hospitals as a setting requiring countermeasures	91.7	3.6
Reporting nightclubs as a setting requiring countermeasures	1.3	91.4
Reporting large indoor events as a setting requiring countermeasures	1.7	75.7
Reporting large outdoor events as a setting requiring countermeasures	1.6	8.9

We conducted a chi-squared test to examine the association between intervention settings and settings reported by participants. We found that more people reported countermeasures being required in care homes, χ2(2) = 3133.48, *p* <.001, and hospitals χ2(2) = 2930.91, *p* <.001, in the health condition than the recreational condition and that more people reported countermeasures being required in nightclubs, χ2(2) = 3037.86, *p* <.001, large indoor events, χ2(2) = 2269.03, *p* <.001, and large outdoor events, χ2(2) = 1268.12, *p* <.001, in the recreational condition than in the health condition.

We conducted a chi-squared test to examine the association between the inclusion of negative LFTs and the expected cost of LFTs and found a significant relationship χ2(10) = 4061.81, *p* <.001.

We conducted a two-way ANOVA to examine whether there were any differences between groups in the ability to visualise the hypothetical scenarios. The results showed that there were no significant differences between the different types of policy, *F* (3,2718) = 0.46, *p* =.71, η_p_^2^ = 0.001, or setting, *F* (1,2718) = 0.04, *p* =.84), η_p_^2^ = < 0.001 nor was there any interaction, *F* (3,2718) = 1.16, *p* =.32, η_p_^2^ = 0.001.

We also conducted exploratory, unregistered, two-way ANOVAs post-unblinding to examine whether there were differences between groups in terms of impact on SDT perceptions. There was a significant effect of policy on autonomy perceptions only, F (3,2718) = 15.17, *p* <.001, η_p_^2^ = 0.016. Bonferroni-corrected post hoc tests indicated higher autonomy scores in the no certification than in the vaccination-only, M*_diff_* = 1.44, 95 % CI [0.79, 2.08], *p* <.001, vaccination status or free LFT, M*_diff_* = 1.03, 95 % CI [0.39, 1.68], *p* <.001, and vaccination status or LFT at cost conditions, M*_diff_* = 1.42, 95 % CI [0.77, 2.06], *p* <.001. The effect of type of policy was not moderated by baseline COVID-19 vaccination status, *F* (3,2718) = 1.53, *p =*.20, R^2^ = 0.0013. There were no effects of setting nor an interaction between setting and policy on any SDT perceptions.

### Impact of COVID-19 certification on behavioural expectations

The means and standard deviations for the baseline and primary outcome measures, by condition, can be found in Table 6, Table 7.

**Table 6:** Means (standard deviation) scores for each baseline score by condition and baseline vaccination status.

**Setting**	**Type of policy**	**Baseline vaccination status**	**Baseline COVID-19 Vaccination Expectation** Mean (Standard Deviation)	**Baseline Flu Vaccination Expectation** Mean (Standard Deviation)	**Baseline Adherence Expectations** Mean (Standard Deviation)
Health	No Certification	Confident	17.76 (3.36)	15.63 (4.09)	10.32 (2.89)
		Hesitant	10.74 (5.69)	11.08 (4.78)	8.88 (3.27)
	Vaccination status	Confident	17.52 (3.60)	16.29 (4.01)	10.37 (3.05)
		Hesitant	10.97 (5.05)	10.91 (4.52)	8.84 (3.34)
	Vaccination status *or* Free Lateral Flow Test	Confident	17.55 (3.58)	16.22 (4.31)	10.40 (3.03)
		Hesitant	11.37 (5.32)	10.64 (4.53)	8.98 (3.28)
	Vaccination status *or* Lateral Flow Test at Cost	Confident	17.66 (3.32)	15.68 (5.30)	10.05 (2.87)
		Hesitant	11.69 (5.54)	11.34 (5.01)	9.20 (3.45)
Recreation	No certification	Confident	17.42 (3.50)	16.33 (4.02)	9.81 (2.90)
		Hesitant	10.83 (5.48)	10.51 (4.80)	8.62 (3.18)
	Vaccination status	Confident	17.93 (2.81)	16.13 (3.96)	10.32 (3.03)
		Hesitant	11.62 (5.67)	11.10 (4.98)	8.86 (3.65)
	Vaccination status *or* Free Lateral Flow Test	Confident	17.66 (3.54)	16.10 (4.31)	10.19 (2.94)
		Hesitant	10.62 (5.16)	10.47 (4.46)	8.81 (3.27)
	Vaccination status *or* Lateral Flow Test at Cost	Confident	17.46 (3.55)	16.10 (4.06)	9.82 (2.99)
		Hesitant	9.79 (5.53)	10.21 (4.99)	8.07 (3.35)

**Table 7:** Means (standard deviation) scores for each primary outcome measure score by condition and baseline vaccination status.

**Setting**	**Type of policy**	**Baseline vaccination status**	**COVID-19 Vaccination Expectation** Mean (Standard Deviation)	**Flu Vaccination Expectation** Mean (Standard Deviation)	**Adherence Expectations** Mean (Standard Deviation)
Health	No certification	Confident	17.92 (3.34)	16.07 (4.07)	13.50 (2.27)
		Hesitant	11.14 (5.83)	11.48 (5.04)	11.50 (3.61)
	Vaccination status	Confident	17.62 (3.54)	16.62 (3.94)	13.44 (2.38)
		Hesitant	11.51 (5.54)	11.04 (4.69)	11.42 (3.93)
	Vaccination status *or* Free Lateral Flow Test	Confident	17.83 (3.48)	16.52 (4.17)	13.65 (2.25)
		Hesitant	11.51 (5.65)	10.60 (4.69)	11.68 (3.70)
	Vaccination status *or* Lateral Flow Test at Cost	Confident	17.82 (3.33)	16.02 (4.29)	13.54 (2.25)
		Hesitant	12.35 (5.77)	11.60 (5.02)	12.11 (3.50)
Recreation	No certification	Confident	17.70 (3.43)	16.63 (4.04)	13.25 (2.54)
		Hesitant	11.33 (5.77)	10.84 (5.14)	11.33 (3.62)
	Vaccination status	Confident	18.07 (3.05)	16.40 (4.05)	13.60 (2.21)
		Hesitant	11.86 (5.73)	11.48 (5.27)	11.30 (3.93)
	Vaccination status *or* Free Lateral Flow Test	Confident	17.82 (3.25)	16.31 (4.16)	13.67 (2.19)
		Hesitant	11.25 (5.60)	10.66 (4.90)	11.16 (3.80)
	Vaccination status *or* Lateral Flow Test at Cost	Confident	17.68 (3.51)	16.40 (4.06)	13.31 (2.67)
		Hesitant	10.28 (5.81)	10.06 (5.24)	10.60 (4.02)

#### Expectations to receive next COVID-19 vaccine

The results of the two way ANCOVA showed that there was no significant effect of setting, F (1,2717) *=* 0.01, *p* =.92, η_p_^2^ < 0.001, type of policy, F (3,2717) = 0.30, *p* =.83, η_p_^2^ < 0.001, nor a significant interaction*,* F (3,2717) = 0.72, *p =*.54*,* η_p_^2^ < 0.001, on expectations to receive one’s next dose of COVID-19 vaccine, after controlling for baseline expectations F (1,2717) = 18282.15, *p* <.001, η_p_^2^ = 0.87.

The moderation analysis, using Model 1 on PROCESS, indicated no interaction between baseline COVID-19 vaccination status (i.e., vaccine confident or hesitant) and setting, F (1,2722) = 1.74, *p =*.19, R^2^ = 0.0004 or baseline vaccination status and type of policy at any level, F (3,2718) = 0.24, *p* =.87, R^2^ = 0.0002., on expectation to receive one’s next dose of the COVID-19 vaccine.

#### Expectations to receive the seasonal flu vaccine

The results of the two way ANCOVA showed that there was no significant effect of setting, F (1,2714) = 0.42, *p* =.52, η_p_^2^ < 0.001, type of policy F (3,2714) *=* 1.59*, p =*.19, η_p_^2^ = 0.002, nor interaction, F (3,2714) *=* 1.04, *p =.*37, η_p_^2^ = 0.003, on expectations to receive the seasonal flu vaccine after controlling for baseline expectations, F (1,2714) = 8720.86, *p* <.001*,* η_p_^2^ = 0.76, receipt of the flu vaccine last winter, F (1,2714) = 15.96 *p* <.001, η_p_^2^ = 0.006, being eligible for a free vaccine, F (1,2714) = 7.10, *p =*.01, η_p_^2^ = 0.003, and being advised to receive the vaccine, F (1,2714) = 0.05, *p =*.82, η_p_^2^ = <0.001.

Moderation analysis, using Model 1 on PROCESS, indicated no interaction between baseline COVID-19 vaccination status (i.e., vaccine confident or hesitant) and setting, F (1,2722) = 2.50 *p =*.11, R^2^ = 0.0007 or baseline vaccination status and policy at any level, F (3,2718) = 0.60, *p* =.61*,* R^2^ = 0.0005 on expectation to receive one’s next dose of the seasonal flu vaccine.

#### Expectations to adhere to protective measures in venues requiring certification^1^

The results of the two way ANCOVA showed that there was no significant effect of setting, F (1,2717) *=* 1.36 2282.27, *p =*.24, η_p_^2^ = 001, type of policy, F (3,2717) *=* 0.52 *p* =.67, η_p_^2^ = 0.001, nor interaction, F (3,2717) *=* 1.37, *p =*.25, η_p_^2^ = 0.002, on expectations to adhere to protective measures in venues requiring such measures, after controlling for baseline expectations F (1,2717) = 2282.274 *p =* < 0.001, η_p_^2^ = 0.46.

Moderation analysis, using Model 1 on PROCESS, did indicate an interaction initially interpreted as significant between baseline COVID-19 vaccination status (i.e., vaccine confident or hesitant) and setting, F (1,2722) = 4.46, *p* =.03, R^2^ = 0.0015, on expectations to adhere to protective measures in venues requiring certification. However, when running the Benjamini-Hochberg procedure, the result was deemed nonsignificant.

There was no significant interaction between baseline vaccination status and type of certification on policy on adherence to protective measures in venues in requiring certification, F (3,2718) = 0.26, *p =*.85, R^2^ = 0.0003.

## Discussion

We carried out an online RCT to assess the impact of different types of COVID-19 certification on subsequent expectations to receive the COVID-19 vaccine, receive the seasonal flu vaccine and adhere to protective behaviours in venues requiring such certification. We hypothesised that, while controlling for baseline COVID-19 vaccine expectations, all types of COVID-19 certification would reduce subsequent behavioural expectations, with expectations being lowest in the vaccination plus LFT at cost condition. We found no significant effects of type or setting of certification on any of the three behavioural expectations.

These findings contrast some findings in the extant literature, which suggest that certification policies can increase uptake of the COVID-19 vaccine [12], [13], [15]. One explanation for this is the high average vaccine uptake in England, as a cross-country analysis reported that countries with below average pre-intervention vaccination uptake show a more profound increase in daily vaccinations compared to those with higher-than-average uptake [13]. In England, uptake of each of the three doses was between 69.3 % and 93.5 % of the population aged over twelve (Table 2), therefore this could explain the limited effect on vaccine uptake. This study also contrasts a previous hypothetical experiment exploring the impact of COVID-19 vaccine mandates on influenza vaccination [30]. This could be due to the timing of the studies as Sprengholz et al’s [30] study was conducted in June 2020 during a time of greater restrictions, whereas the present study was conducted after the cessation of COVID-19 restrictions.

This study also hypothesised that the effect of certification would be moderated by baseline vaccine status, with effects being present in the vaccine hesitant cohort and not the vaccine confident cohort. We found no significant moderating effect; in both groups there was no significant effect of certification type or setting on any behavioural expectation. These findings contrast previous studies reporting that the impact of certification on vaccine intentions may be greater amongst vaccine hesitant individuals [10], [19]. This could be explained by the different study designs and contexts. The present study was an intervention design conducted after the implementation of COVID-19 certification, whereas the previous studies were conducted before the implementation of COVID-19 certification.

As part of an unregistered exploratory analysis, we examined the impact of certification on the constructs within SDT and found that all types of certification increased autonomy frustration compared to no certification. This supports previous interview studies highlighting concerns associated with the impact of vaccine certification on autonomy [20], [24], [25] and survey results suggesting an association of autonomy frustration with a reduction in self-reported willingness to receive the COVID-19 vaccine and self-reported uptake of the vaccine [22]. However, this study suggests that although certification may increase autonomy frustration, it does not necessarily translate into a reduction in uptake of the COVID-19 vaccine.

### Implications and recommendations

If the decision to implement certification is underpinned by intent to increase vaccination uptake, alternative interventions such as personalised communication or being open about uncertainties may be more effective than certification [46]. Similarly, to increase uptake of the seasonal flu vaccine, campaigns should target underlying barriers, such as low perceived risk and lack of knowledge [47]. However, increased vaccine uptake is not always the intent of certification. For example, certification may be used to control transmission or reduce serious infection. In this case, this study would suggest that certification would not necessarily have a negative spill-over effect on vaccination, even amongst vaccine hesitant individuals. However, these findings should be corroborated with studies on the effect of certification on objective vaccination uptake data before conclusions can be drawn regarding the overall risks and benefits of certification. These findings also suggest that certification would not necessarily offset the potential reduction in transmission by increasing non-adherence to non-pharmaceutical interventions (NPIs), although it is important to corroborate this with assessment of observed adherence to NPIs in settings requiring certification.

### Strengths and limitations

This study is novel in exploring the behavioural effects of variations in COVID-19 certification using an experimental design. A limitation of this research is that it used a hypothetical scenario and behavioural expectations, which may not be linked to actual behaviours. Future research could benefit from observational methods, particularly to examine adherence to protective behaviours, to see if there are any differences in adherence across different settings. Similarly, this study used self-report data, so it is important to interpret these findings alongside the findings from studies using objective vaccine uptake data in non-hypothetical scenarios to gain a broader understanding of the behavioural impact of certification (e.g., [13]). Also, as all domestic COVID-19 restrictions in England were removed six months prior to the experiment with no current discussion about their reintroduction, it may have felt more hypothetical than if it was conducted before its implementation. Another limitation is that the word count and readability scores were not consistent between conditions. However, unless new confounds were added into the control conditions, this was unavoidable. Furthermore, experimental designs can be reductionist as many factors influence vaccine uptake including perceived vaccine safety and effectiveness [48], trust in the government [11] and history of influenza or COVID-19. Similarly, participants have limited time to process the scenarios during the experiment, compared to the extended time to consider real-world policies. There is also the potential for selection bias as it is possible that there are fundamental differences in the characteristics of individuals who are members of online participant pools and those who are not. It is therefore important that these factors are also taken into consideration.

### Future research

Whilst this study explored the behavioural impact of types of certification and settings in which certification is required, other variations of certification that could be explored in the future include who sets the certification requirement (e.g., government rules or venue rules) and what is the stated intent of certification (e.g., increase vaccine uptake, reduce transmission, or increase testing). Future research could explore this using behavioural interventions or qualitative research to understand the circumstances in which the public would feel differently towards certification and quasi-mandatory vaccination. Additionally, this study categorised people as vaccine hesitant if they had two or fewer vaccines; future research could explore whether there are differences in the effect of certification within the vaccine hesitant cohort.

### Conclusion

This study explored the impact of different types of COVID-19 certification on subsequent behavioural expectations. We found no differences in expectations to receive the next dose of the COVID-19 vaccine, to receive the seasonal flu vaccine or to adhere to protective measures between any of our certification conditions. The study also found no differences in such outcomes between vaccine hesitant participants and vaccine confident participants. Considering these findings, COVID-19 certification is unlikely to offset transmission risk through increased risky behaviour and is unlikely to have a negative spill-over effect in decreasing COVID-19 and seasonal flu vaccinations, even amongst vaccine hesitant individuals. However, if the intention of certification is to increase vaccine uptake, alternative interventions should be sought. Although these findings provide an insight into the behavioural effects of COVID-19 certification, they should be interpreted with caution as they are based on a hypothetical scenario and behavioural expectations (as opposed to observed or reported behaviour) following the cessation of domestic COVID-19 certification and restrictions.

**Pre-registered protocol:**https://osf.io/8dhn6.

**Statement of contribution:**

What is already known on the topic?

• COVID-19 certification could increase COVID-19 vaccine uptake.
• Myriad of factors influence impact of certification on COVID-19 vaccine uptake, e.g., vaccine hesitancy.
• A key concern of certification is loss of individual autonomy.

What does this study add?

• Indicates that behavioural expectations do not change when COVID-19 certification is an additional entry requirement.
• Indicates that, behavioural expectations do not vary between different types of COVID-19 certifications.
• Indicates that baseline vaccination status does not moderate the relationship between COVID-19 certification and expectations.

## Funding statement

HC and FM are supported by the National Institute for Health Research Health Protection Research Unit (NIHR HPRU) in Emergency Preparedness and Response, a partnership between UK Health Security Agency (UKHSA), King’s College London and the University of East Anglia. The views expressed are those of the authors and not necessarily those of the NIHR, UKHSA or the Department of Health and Social Care. All authors had full access to the data and can take responsibility for the integrity of the data and the accuracy of the data analysis. No external funding organisation had a role in the design of the study; in the collection, analyses, or interpretation of data; in the writing of the manuscript, or in the decision to publish the results.

## Declaration of Competing Interest

The authors declare that they have no known competing financial interests or personal relationships that could have appeared to influence the work reported in this paper.

## Data Availability

Data will be made available on reasonable request.

## Footnotes

1 The results of the moderation analyses held when factoring in the same covariates entered into the ANCOVA models for RQs 1–3. As an extra precaution we addressed RQ4 by splitting the data according to baseline vaccination status (confident or hesitant) and running the same ANCOVAs as used to address RQs1-3 and the results were consistent with the results of moderation analysis reported below.
