# Supplementary material for: An online RCT on behavioural expectations effects of COVID-19 certification policies in England
Source: Vaccine X. 2023 Sep 20;15:100389. doi: 10.1016/j.jvacx.2023.100389 (PMC10565557; doi:10.1016/j.jvacx.2023.100389)
Supplement: Supplementary Data 2 [file mmc2.docx]

**Online RCT on Behavioural Expectations Effects of COVID-19 Certification Policies in England**

**Supplementary File 2 - Scenarios**

Randomised scenarios [please note, participants each received one of these scenarios]

Scenario 1: no certification in healthcare settings

**Please read the following information and picture yourself in the situation described below.**

**Please pay close to attention as you will be asked questions on this information later on in the study.**

Imagine that the following announcement has just been made: “Due to the rise in COVID-19 infections in England, new measures are being introduced. From next week, face coverings and social distancing are required for entry into care homes and hospitals, unless exempt. It is also advised that you receive your next dose of the COVID-19 vaccine when offered. You can book your vaccine appointment on gov.uk.”

Scenario 2: vaccination status only certification in healthcare settings

**Please read the following information and picture yourself in the situation described below.**

**Please pay close to attention as you will be asked questions on this information later on in the study.**

Imagine that the following announcement has just been made: “Due to the rise in COVID-19 infections in England, new measures are being introduced. From next week, face coverings, social distancing and an NHS COVID Pass are required for entry into care homes and hospitals, unless exempt. Individuals will have to demonstrate that they have received at least three doses of the COVID-19 vaccine to access these sites. You can book your vaccine appointment on gov.uk.”

Scenario 3: vaccination status or free testing in healthcare settings

**Please read the following information and picture yourself in the situation described below.**

**Please pay close to attention as you will be asked questions on this information later on in the study.**

Imagine that the following announcement has just been made: “Due to the rise in COVID-19 infections in England, new measures are being introduced. From next week, face coverings, social distancing and an NHS COVID Pass are required for entry into care homes and hospitals, unless exempt. Individuals will have to demonstrate that they have received at least three doses of the COVID-19 vaccine or have had a negative Lateral Flow test in the last 48 hours. You can book your vaccine appointment and order your free Lateral Flow tests on gov.uk or pick the tests up from a pharmacy near you.”

Scenario 4: vaccination status or testing at cost in healthcare settings

**Please read the following information and picture yourself in the situation described below.** 
**Please pay close to attention as you will be asked questions on this information later on in the study.**

Imagine that the following announcement has just been made: “Due to the rise in COVID-19 infections in England, new measures are being introduced. From next week, face coverings, social distancing and an NHS COVID Pass are required for entry into care homes and hospitals, unless exempt. Individuals will have to demonstrate that they have received at least three doses of the COVID-19 vaccine or have had a negative Lateral Flow test in the last 48 hours. You can book your vaccine appointment on gov.uk or purchase Lateral Flow tests from a pharmacy near you.”

Scenario 5: no certification in recreational settings

**Please read the following information and picture yourself in the situation described below.**

**Please pay close to attention as you will be asked questions on this information later on in the study.**

Imagine that the following announcement has just been made: “Due to the rise in COVID-19 infections in England, new measures are being introduced. From next week, face coverings and social distancing are required for entry into nightclubs and large indoor and outdoor events, unless exempt. It is also advised that you receive your next dose of the COVID-19 vaccine when offered. You can book your vaccine appointment on gov.uk.”

Scenario 6: vaccination status only certification in recreational settings

**Please read the following information and picture yourself in the situation described below.**

**Please pay close to attention as you will be asked questions on this information later on.**

Imagine that the following announcement has just been made: “Due to the rise in COVID-19 infections in England, new measures are being introduced. From next week, face coverings, social distancing and an NHS COVID Pass are required for entry into nightclubs and large indoor and outdoor events, unless exempt. Individuals will have to demonstrate that they have received at least three doses of the COVID-19 vaccine. You can book your vaccine appointment on gov.uk.”

Scenario 7: vaccination status or free testing in recreational settings

**Please read the following information and picture yourself in the situation described below.**

**Please pay close to attention as you will be asked questions on this information later on.**

Imagine that the following announcement has just been made: “Due to the rise in COVID-19 infections in England, new measures are being introduced. From next week, face coverings, social distancing and an NHS COVID Pass are required for entry into nightclubs and large indoor and outdoor events, unless exempt. Individuals will have to demonstrate that they have received at least three doses of the COVID-19 vaccine or have had a negative Lateral Flow test in the last 48 hours. You can book your vaccine appointment and order your free Lateral Flow tests on gov.uk or pick the tests up from a pharmacy near you.”

Scenario 8: vaccination status or testing at cost in recreational settings

**Please read the following information and picture yourself in the situation described below.**

**Please pay close to attention as you will be asked questions on this information later on.**

Imagine that the following announcement has just been made: “Due to the rise in COVID-19 infections in England, new measures are being introduced. From next week, face coverings, social distancing and an NHS COVID Pass are required for entry into nightclubs and large indoor and outdoor events, unless exempt. Individuals will have to demonstrate that they have received at least three doses of the COVID-19 vaccine or have had a negative Lateral Flow test in the last 48 hours. You can book your vaccine appointment on gov.uk or purchase Lateral Flow tests from a pharmacy near you.”
