# Supplementary material for: An online RCT on behavioural expectations effects of COVID-19 certification policies in England
Source: Vaccine X. 2023 Sep 20;15:100389. doi: 10.1016/j.jvacx.2023.100389 (PMC10565557; doi:10.1016/j.jvacx.2023.100389)
Supplement: Supplementary Data 3 [file mmc3.docx]

**Online RCT on Behavioural Expectations Effects of COVID-19 Certification Policies in England**

**Supplementary File 3 - Questions**

The below presents the experiment and questionnaire, as viewed by the participants after completing the consent form. Please note, participants did not view the headings presented below.

Baseline COVID-19 vaccination behaviour

- Please indicate which describes your current situation:
  - I have not had the COVID-19 vaccine and I have not booked an appointment to have it
  - I have not had the COVID-19 vaccine and I have booked an appointment to have the first dose
  - I have had one dose of the COVID-19 vaccine and I have not booked an appointment to have the second dose
  - I have had one dose of the COVID-19 vaccine and I have booked an appointment to have the second dose
  - I have had two doses of the COVID-19 vaccine and I have not booked an appointment to have the third dose
  - I have had two doses of the COVID-19 vaccine and I have booked an appointment to have the third dose
  - I have had three doses of the COVID-19 vaccine and I have not booked an appointment to have the fourth dose
  - I have had three doses of the COVID-19 vaccine and I have booked an appointment to have the fourth dose
  - I have had four doses of the COVID-19 vaccine

The following questions relate to the COVID-19 vaccine. Please rate how likely or unlikely you would be to take each of the following actions.

- How likely or unlikely would you be to get the next dose of the COVID-19 vaccine offered to you?
  - 1 (very unlikely) – 5 (very likely)
- How likely or unlikely would you be to recommend getting the COVID-19 vaccine to someone who seeks your advice?
  - 1 (very unlikely) – 5 (very likely)
- How likely or unlikely would you be to encourage your friends and relatives to get the COVID-19 vaccine?
  - 1 (very unlikely) – 5 (very likely)
- How likely or unlikely would you be to say positive things about getting the COVID-19 vaccine?
  - 1 (very unlikely) – 5 (very likely)

*Baseline other vaccination behaviour*

- Were you advised to get the flu vaccine last winter (e.g. by your GP)?
  - Yes
  - No
  - Don’t know
- Are you eligible for a free annual flu vaccine?
  - Yes
  - No
  - Don’t know
- Please indicate which best describes your current situation:
  - I received the flu vaccine last winter
  - I did not receive the flu vaccine last winter

Please rate how likely or unlikely you would be to take each of the following actions.

- How likely or unlikely would you be to get the seasonal flu vaccine this winter?
  - 1 (very unlikely) – 5 (very likely)
- How likely or unlikely would you be to recommend getting the seasonal flu vaccine to someone who seeks your advice?
  - 1 (very unlikely) – 5 (very likely)
- How likely or unlikely would you be to encourage your friends and relatives to get the seasonal flu vaccine?
  - 1 (very unlikely) – 5 (very likely)
- How likely or unlikely would you be to say positive things about getting the seasonal flu vaccine?
  - 1 (very unlikely) – 5 (very likely)

Baseline Adherence to protective behaviours in settings

- In future, how likely or unlikely are you to wear a face covering in public settings?
  - 1 (very unlikely) – 5 (very likely)
- In future, how likely or unlikely are you to apply hand sanitiser in public settings?
  - 1 (very unlikely) – 5 (very likely)
- In future, how likely or unlikely are you to attempt to keep two metres apart from people outside your household in public settings?
  - 1 (very unlikely) – 5 (very likely)

*Randomised scenario* [please note, participants only received one of these scenarios]

- Scenario 1: no certification in healthcare settings
  - **Please read the following information and picture yourself in the situation described below.**

**Please pay close to attention as you will be asked questions on this information later on in the study.**

Imagine that the following announcement has just been made: “Due to the rise in COVID-19 infections in England, new measures are being introduced. From next week, face coverings and social distancing are required for entry into care homes and hospitals, unless exempt. It is also advised that you receive your next dose of the COVID-19 vaccine when offered. You can book your vaccine appointment on gov.uk.”

- Scenario 2: vaccination status only certification in healthcare settings
  - **Please read the following information and picture yourself in the situation described below.**

**Please pay close to attention as you will be asked questions on this information later on in the study.**

Imagine that the following announcement has just been made: “Due to the rise in COVID-19 infections in England, new measures are being introduced. From next week, face coverings, social distancing and an NHS COVID Pass are required for entry into care homes and hospitals, unless exempt. Individuals will have to demonstrate that they have received at least three doses of the COVID-19 vaccine to access these sites. You can book your vaccine appointment on gov.uk.”

- Scenario 3: vaccination status or free testing in healthcare settings
  - **Please read the following information and picture yourself in the situation described below.**

**Please pay close to attention as you will be asked questions on this information later on in the study.**

Imagine that the following announcement has just been made: “Due to the rise in COVID-19 infections in England, new measures are being introduced. From next week, face coverings, social distancing and an NHS COVID Pass are required for entry into care homes and hospitals, unless exempt. Individuals will have to demonstrate that they have received at least three doses of the COVID-19 vaccine or have had a negative Lateral Flow test in the last 48 hours. You can book your vaccine appointment and order your free Lateral Flow tests on gov.uk or pick the tests up from a pharmacy near you.”

- Scenario 4: vaccination status or testing at cost in healthcare settings
  - **Please read the following information and picture yourself in the situation described below.** 
    **Please pay close to attention as you will be asked questions on this information later on in the study.**

Imagine that the following announcement has just been made: “Due to the rise in COVID-19 infections in England, new measures are being introduced. From next week, face coverings, social distancing and an NHS COVID Pass are required for entry into care homes and hospitals, unless exempt. Individuals will have to demonstrate that they have received at least three doses of the COVID-19 vaccine or have had a negative Lateral Flow test in the last 48 hours. You can book your vaccine appointment on gov.uk or purchase Lateral Flow tests from a pharmacy near you.”

- Scenario 5: no certification in recreational settings
  - **Please read the following information and picture yourself in the situation described below.**

**Please pay close to attention as you will be asked questions on this information later on in the study.**

Imagine that the following announcement has just been made: “Due to the rise in COVID-19 infections in England, new measures are being introduced. From next week, face coverings and social distancing are required for entry into nightclubs and large indoor and outdoor events, unless exempt. It is also advised that you receive your next dose of the COVID-19 vaccine when offered. You can book your vaccine appointment on gov.uk.”

- Scenario 6: vaccination status only certification in recreational settings
  - **Please read the following information and picture yourself in the situation described below.**

**Please pay close to attention as you will be asked questions on this information later on.**

Imagine that the following announcement has just been made: “Due to the rise in COVID-19 infections in England, new measures are being introduced. From next week, face coverings, social distancing and an NHS COVID Pass are required for entry into nightclubs and large indoor and outdoor events, unless exempt. Individuals will have to demonstrate that they have received at least three doses of the COVID-19 vaccine. You can book your vaccine appointment on gov.uk.”

- Scenario 7: vaccination status or free testing in recreational settings
  - **Please read the following information and picture yourself in the situation described below.**

**Please pay close to attention as you will be asked questions on this information later on.**

Imagine that the following announcement has just been made: “Due to the rise in COVID-19 infections in England, new measures are being introduced. From next week, face coverings, social distancing and an NHS COVID Pass are required for entry into nightclubs and large indoor and outdoor events, unless exempt. Individuals will have to demonstrate that they have received at least three doses of the COVID-19 vaccine or have had a negative Lateral Flow test in the last 48 hours. You can book your vaccine appointment and order your free Lateral Flow tests on gov.uk or pick the tests up from a pharmacy near you.”

- Scenario 8: vaccination status or testing at cost in recreational settings
  - **Please read the following information and picture yourself in the situation described below.**

**Please pay close to attention as you will be asked questions on this information later on.**

Imagine that the following announcement has just been made: “Due to the rise in COVID-19 infections in England, new measures are being introduced. From next week, face coverings, social distancing and an NHS COVID Pass are required for entry into nightclubs and large indoor and outdoor events, unless exempt. Individuals will have to demonstrate that they have received at least three doses of the COVID-19 vaccine or have had a negative Lateral Flow test in the last 48 hours. You can book your vaccine appointment on gov.uk or purchase Lateral Flow tests from a pharmacy near you.”

*Visualisation*:

The following questions refer to the information you have just read.

- I was able to imagine this situation well
  - 1 (strongly disagree) – 5 (strongly agree)
- I was able to emotionally engage with this situation
  - 1 (strongly disagree) – 5 (strongly agree)

*Attention check*

- How worried would we be if you didn’t pay attention? To check that you are paying attention, please do not select an answer below
  - 1 (strongly disagree) – 5 (strongly agree)

#### COVID-19 vaccination expectation

The questions in this section are relating to the COVID-19 vaccine. Please rate how likely or unlikely you would be to take each of the following actions following this announcement.

- If this announcement were real, how likely or unlikely would you be to get the next dose of the COVID-19 vaccine offered to you?
  - 1 (very unlikely) – 5 (very likely)
- If this announcement were real, how likely or unlikely would you be to recommend getting the COVID-19 vaccine to someone who seeks your advice?
  - 1 (very unlikely) – 5 (very likely)
- If this announcement were real, how likely or unlikely would you be to encourage your friends and relatives to get the COVID-19 vaccine?
  - 1 (very unlikely) – 5 (very likely)
- If this announcement were real, how likely or unlikely would you be to say positive things about getting the COVID-19 vaccine?
  - 1 (very unlikely) – 5 (very likely)

#### Other vaccination expectation

The questions in this section are relating to vaccines other than COVID-19. Please rate how likely or unlikely you would be to take each of the following actions following this announcement.

- If this announcement were real, how likely or unlikely would you be to get the seasonal flu vaccine this winter?
  - 1 (very unlikely) – 5 (very likely)
- If this announcement were real, how likely or unlikely would you be to recommend getting the seasonal flu vaccine to someone who seeks your advice?
  - 1 (very unlikely) – 5 (very likely)
- If this announcement were real, how likely or unlikely would you be to encourage your friends and relatives to get the seasonal flu vaccine?
  - 1 (very unlikely) – 5 (very likely)
- If this announcement were real, how likely or unlikely would you be to say positive things about getting the seasonal flu vaccine?
  - 1 (very unlikely) – 5 (very likely)

Adherence to protective behaviours in settings

- If this announcement were real, how likely or unlikely are you to take the following actions in venues requiring these COVID-19 measures?
  - Wear a face covering
    - 1 (very unlikely) – 5 (very likely)
  - Apply hand sanitiser when entering the venues
    - 1 (very unlikely) – 5 (very likely)
  - Attempt to keep two metres apart from people outside your household
    - 1 (very unlikely) – 5 (very likely)

*Use of settings for which certification would be applied*

- [For individuals in healthcare settings] Do you work in a hospital or care home?
  - Yes
  - No
- [For individuals in recreational settings] Do you work in a nightclub or at large indoor or outdoor events?
  - Yes
  - No
- How often do you visit these settings?
  - Never, rarely, sometimes, often, always

*Self-determination Theory*

Following this announcement, consider the information and rate your agreement with the following statements.

- Autonomy satisfaction:
  - - I would feel a sense of choice and freedom in the decision to get the COVID-19 vaccine in future
      - - 1 (strongly disagree) – 5 (strongly agree)
    - I would feel that my decision to get the COVID-19 vaccine in future reflects what I really want
      - - 1 (strongly disagree) – 5 (strongly agree)
    - I would feel a sense of choice and freedom in the decision to wear a face covering in the future
      - - 1 (strongly disagree) – 5 (strongly agree)
    - I would feel that my decision to wear a face covering in the future reflects what I really want
      - - 1 (strongly disagree) – 5 (strongly agree)
    - I would feel a sense of choice and freedom in the decision to socially distance myself from others in the future
      - - 1 (strongly disagree) – 5 (strongly agree)
    - I would feel that my decision to socially distance myself from others in the future reflects what I really want
      - - 1 (strongly disagree) – 5 (strongly agree)
- Autonomy frustration
  - - I would feel forced to get the COVID-19 vaccine
      - - 1 (strongly disagree) – 5 (strongly agree)
    - I would feel that I would be discriminated against if I didn’t get the COVID-19 vaccine
      - - 1 (strongly disagree) – 5 (strongly agree)
    - I would feel forced to wear a face covering
      - - 1 (strongly disagree) – 5 (strongly agree)
    - I would feel that I would be discriminated against if I didn’t wear a face covering
      - - 1 (strongly disagree) – 5 (strongly agree)
    - I would feel forced to socially distance myself from others
      - - 1 (strongly disagree) – 5 (strongly agree)
    - I would feel that I would be discriminated against if I didn’t socially distance myself from others
      - - 1 (strongly disagree) – 5 (strongly agree)
- Competence satisfaction
  - - I would feel confident that I could get the COVID-19 vaccine if I wanted to
      - - 1 (strongly disagree) – 5 (strongly agree)
    - I would feel capable of getting the COVID-19 vaccine if I wanted to
      - - 1 (strongly disagree) – 5 (strongly agree)
    - I would feel confident that I could wear a face covering if I wanted to
      - - 1 (strongly disagree) – 5 (strongly agree)
    - I would feel capable of wearing a face covering if I wanted to
      - - 1 (strongly disagree) – 5 (strongly agree)
    - I would feel confident that I could socially distance myself from others if I wanted to
      - - 1 (strongly disagree) – 5 (strongly agree)
    - I would feel capable of socially distancing myself from others if I wanted to
      - - 1 (strongly disagree) – 5 (strongly agree)
- Competence frustration
  - - I would have serious doubts about whether I could get the COVID-19 vaccine if I wanted to
      - - 1 (strongly disagree) – 5 (strongly agree)
    - I would feel that it would be difficult for me to get the COVID-19 vaccine if I wanted to
      - - 1 (strongly disagree) – 5 (strongly agree)
    - I would have serious doubts about whether I could wear a face covering if I wanted to
      - - 1 (strongly disagree) – 5 (strongly agree)
    - I would feel that it would be difficult for me to wear a face covering if I wanted to
      - - 1 (strongly disagree) – 5 (strongly agree)
    - I would have serious doubts about whether I could socially distance myself from others if I wanted to
      - - 1 (strongly disagree) – 5 (strongly agree)
    - I would feel that it would be difficult for me to socially distance myself from others if I wanted to
      - - 1 (strongly disagree) – 5 (strongly agree)
- Relatedness satisfaction
  - - I would feel that the government implementing these measures cares about what is best for me
      - - 1 (strongly disagree) – 5 (strongly agree)
    - I would feel that the government implementing these measures understands my beliefs towards the COVID-19 vaccination
      - - 1 (strongly disagree) – 5 (strongly agree)
- Relatedness frustration
  - - I would feel excluded by the government implementing these measures
      - - 1 (strongly disagree) – 5 (strongly agree)
    - I would feel that the government implementing these measures did not understand how I feel about the COVID-19 vaccination
      - - 1 (strongly disagree) – 5 (strongly agree)

*Manipulation check:*

- Which measures are required for entry to the settings mentioned? Please select all that apply [please note, these options were randomised for participants]
  - Wear a face covering
  - Wash hands upon entry
  - Social distancing
  - Three doses of a COVID-19 vaccine
  - Negative Lateral Flow test
  - Maximum group size of 6 people
- [For those in the two certification-or-testing conditions] How much do you think a Lateral Flow test in this announcement would cost you personally?
  - Free
  - £1 - £2 per test
  - £2- £5 per test
  - £5 - £10 per test
  - More than £10 per test
- Which settings were mentioned for requiring these new measures? [please note, these options were randomised for participants]
  - Hospitals
  - Care homes
  - Night clubs
  - Sporting events
  - Music events
  - Restaurants
  - Public transport
  - Large indoor events
  - Large outdoor events

Vaccination Concerns in COVID-19 Scale

The below questions relate to the **COVID-19 vaccine.** Please rate your agreement with the following statements:

*Beliefs in vaccine efficacy and prevention*

- If I get the COVID-19 vaccine it will help to protect my family and friends against the coronavirus
  - 1 (strongly disagree) – 5 (strongly agree)
- The COVID-19 vaccine will protect me from the coronavirus
  - 1 (strongly disagree) – 5 (strongly agree)
- The COVID-19 vaccine will stop the spread of the coronavirus
  - 1 (strongly disagree) – 5 (strongly agree)
- The COVID-19 vaccine is effective
  - 1 (strongly disagree) – 5 (strongly agree
- The COVID-19 vaccine will reduce the severity of symptoms if I get the coronavirus
  - 1 (strongly disagree) – 5 (strongly agree)
- Getting the COVID-19 vaccine will help to get things back to normal
  - 1 (strongly disagree) – 5 (strongly agree
- It is important to get the COVID-19 vaccine so that outbreaks do not occur
  - 1 (strongly disagree) – 5 (strongly agree)
- Getting the COVID-19 vaccine is important for the health of others in my community
  - 1 (strongly disagree) – 5 (strongly agree)

*Trust in authorities*

- I trust the UK government to give me reliable information on the benefits and risks of the COVID-19 vaccine
  - 1 (strongly disagree) – 5 (strongly agree)
- I trust healthcare providers and health professionals to give me reliable information on the benefits and risks of the COVID-19 vaccine
  - 1 (strongly disagree) – 5 (strongly agree)
- I trust the UK government’s conclusions that the COVID-19 vaccine is safe
  - 1 (strongly disagree) – 5 (strongly agree)
- I trust scientists to give me reliable information on the benefits and risks of the COVID-19 vaccine
  - 1 (strongly disagree) – 5 (strongly agree)
- I trust scientists’ conclusions that the COVID-19 vaccine is safe
  - 1 (strongly disagree) – 5 (strongly agree)
- I trust healthcare providers’ and health professionals’ conclusions that the COVID-19 vaccine is safe
  - 1 (strongly disagree) – 5 (strongly agree)
- I trust the vaccine manufacturers to give me reliable information on the benefits and risks of the COVID-19 vaccine
  - 1 (strongly disagree) – 5 (strongly agree)

*Worry about safety and side-effects*

- I am concerned about the side-effects of the COVID-19 vaccine
  - 1 (strongly disagree) – 5 (strongly agree)
- I fear that the COVID-19 vaccine will cause side effects
  - 1 (strongly disagree) – 5 (strongly agree)
- I am worried about the safety of the COVID-19 vaccine
  - 1 (strongly disagree) – 5 (strongly agree)
- I am

*Beliefs that the vaccine causes COVID-19*

- The COVID-19 vaccine can cause the coronavirus in some people
  - 1 (strongly disagree) – 5 (strongly agree)
- The COVID-19 vaccine can give you a serious case of the very same virus you’re trying to avoid
  - 1 (strongly disagree) – 5 (strongly agree)
- I can get the coronavirus from the COVID-19 vaccine
  - 1 (strongly disagree) – 5 (strongly agree)

*Scepticism and mistrust in the vaccine*

- The COVID-19 vaccine safety data is often made up
  - 1 (strongly disagree) – 5 (strongly agree)
- People have been deceived about the safety of the COVID-19 vaccine
  - 1 (strongly disagree) – 5 (strongly agree)
- The COVID-19 vaccine is promoted mainly because of manufactures profits
  - 1 (strongly disagree) – 5 (strongly agree)
- The main reason for promoting the COVID-19 vaccine is for drug companies to make money
  - 1 (strongly disagree) – 5 (strongly agree)
- I am opposed to the COVID-19 vaccine because it goes against freedom of choice
  - 1 (strongly disagree) – 5 (strongly agree)

*Fear of the vaccine*

- I am afraid of getting the COVID-19 vaccine
  - 1 (strongly disagree) – 5 (strongly agree)
- I am fearful about getting the COVID-19 vaccine
  - 1 (strongly disagree) – 5 (strongly agree)
- Getting the COVID-19 vaccine makes me feel anxious
  - 1 (strongly disagree) – 5 (strongly agree)

*Uncertainty and hesitation in getting vaccinated*

- The COVID-19 vaccine is too new so I should wait before deciding to get it
  - 1 (strongly disagree) – 5 (strongly agree)
- More time is needed to be able to fully investigate the true effects of the COVID-19 vaccine
  - 1 (strongly disagree) – 5 (strongly agree)
- I am afraid that the COVID-19 vaccine has not been successfully tested on enough people
  - 1 (strongly disagree) – 5 (strongly agree)

*Vaccine literacy*

- I have access to all the information I need to make good decisions about getting the COVID-19 vaccine
  - 1 (strongly disagree) – 5 (strongly agree)
- Information about the COVID-19 vaccine is easy to understand
  - 1 (strongly disagree) – 5 (strongly agree)
- I don’t have enough information about the COVID-19 vaccine to decide
  - 1 (strongly disagree) – 5 (strongly agree)

*Adult Vaccine Hesitancy Scale*

The following questions relate to **vaccines in general** rather than the COVID-19 vaccine. Please rate your agreement with these statements:

- Vaccines are important for my health
  - 1 (strongly disagree) – 5 (strongly agree)
- Vaccines are effective
  - 1 (strongly disagree) – 5 (strongly agree)
- Being vaccinated is important for the health of others in my community
  - 1 (strongly disagree) – 5 (strongly agree)
- All routine vaccinations recommended by the NHS are beneficial
  - 1 (strongly disagree) – 5 (strongly agree)
- New vaccines carry more risks than older vaccines
  - 1 (strongly disagree) – 5 (strongly agree)
- The information received about the vaccines from the NHS are reliable and trustworthy
  - 1 (strongly disagree) – 5 (strongly agree)
- Getting vaccinated is a good way to protect me from disease
  - 1 (strongly disagree) – 5 (strongly agree)
- Generally, I do what doctors or health care providers recommend about vaccines
  - 1 (strongly disagree) – 5 (strongly agree)
- I am concerned about serious adverse effects of vaccines
  - 1 (strongly disagree) – 5 (strongly agree)
- I do not need vaccines for diseases that are not common anymore
  - 1 (strongly disagree) – 5 (strongly agree)

*Attention check*

- The colour test you are about to take part in is very simple, when asked for your favourite colour you must select ‘Blue’. This is an attention check.

Based on the text you read above, what colour have you been asked to enter?

- - Red; Blue; Purple; Brown; Orange

*Demographics*

Please note the following questions are voluntary.

*Age*

- What was your age last birthday? [open-ended number response]

*Gender*

- What is your sex?
  - Options: Female; Male; Prefer not to say
- Is the gender you identify with the same as your sex registered at birth
  - Options: Yes; No (Enter gender identity….); prefer not to say

*Ethnicity*

- What is your ethnic group? [Choose one option that best describes your ethnic group or background]
  - White
    - English/Welsh/Scottish/Northern Irish/British
    - Irish
    - Gypsy or Irish Traveller
    - Any other White background, please describe
  - Mixed/Multiple ethnic groups
    - White and Black African
    - White and Asian
    - White and Black Caribbean
    - Any other Mixed/Multiple ethnic background, please describe
  - Asian/Asian British
    - Indian
    - Pakistani
    - Bangladeshi
    - Chinese
    - Any other Asian background, please describe
  - Black/ African/Caribbean/Black British
    - African
    - Caribbean
    - Any other Black/African/Caribbean background, please describe
  - Other ethnic group
    - Arab
    - Any other ethnic group, please describe
  - Prefer not to say

*Education*

- What is your highest educational qualification achieved to date? [If you are currently working towards a qualification, please select this option from the list below]
- No qualifications
- GCSE , O-Level or equivalent
- A-Level, AS-Level, or equivalent
- Professional qualification
- Trade apprenticeship
- Undergraduate degree
- Postgraduate degree or higher
- Other UK qualification (please describe)
- Other non-UK qualification (please describe)
- Prefer not to say

*Geographical location*

- In which part of the UK are you currently based?
  - South East England
  - South West England
  - East Anglia
  - London
  - East Midlands
  - West Midlands
  - North West England
  - North East England
  - Yorkshire & Humber
  - Prefer not to say

*Employment status*

- Which of these options best describes your current situation?
  - - Full-time employed
    - Part-time employed
    - Self-employed (full or part-time)
    - Fulltime education (including part-time or seasonal employment whilst in full-time education)
    - In paid work with a zero-hours contract
    - Not in paid work
    - On a government supported training programme
    - Employed (full or parttime) and on long-term leave
    - Retired
    - Other (please describe)
    - Prefer not to say

*Income*

- Which of the following categories best describes your ANNUAL household income (total income before taxes, or gross income, of all persons in your household combined, for one year)
  - - Under £6,500
    - £6,501-15,000
    - £15,001-30,000
    - £30,001-40,000
    - £40,001-50,000
    - £50,001-65,000
    - £65,001-95,000
    - £95,001 and over
    - Don’t know
    - Prefer not to say

[Participants were then presented with the debriefing statement.]

If you would like to receive a copy of the results from this study, please click yes in the box below. Once the study has been completed, we will send you a link to the report through Prolific’s anonymous messaging service.
