# Supplementary material for: An online RCT on behavioural expectations effects of COVID-19 certification policies in England
Source: Vaccine X. 2023 Sep 20;15:100389. doi: 10.1016/j.jvacx.2023.100389 (PMC10565557; doi:10.1016/j.jvacx.2023.100389)
Supplement: Supplementary Data 4 [file mmc4.docx]

**Online RCT on Behavioural Expectations Effects of COVID-19 Certification Policies in England**

**Supplementary File 4 – Blinding procedures**

On completion of all data collection, FM transferred the data to a secure location accessible by CS, who substituted all level labels for Policy, Setting and Baseline COVID-19 vaccination status with alphanumeric codes obtained from randomcodegenerator.com. It was not possible to apply blinding to the name of the independent variables because the analyst was already aware that there are a different number of levels for each variable. It was also necessary to label the “no certification” label in the policy condition as “Control” because the analyst needed to use the control condition as the reference point for moderation and mediation analyses in which Policy is the independent variable due to the number of levels of this variable exceeding two. Therefore, the only labels that could be concealed to the analyst during analysis were the Setting and Baseline COVID-19 vaccination status levels and experimental levels in the Policy variable.

Responses to the question pertaining to COVID-19 vaccination status at baseline, the treatment arm to which participants were randomly allocated, and the score for manipulation checks following coding (e.g. “fully correct for *x* condition”) were also coded to prevent accidental unblinding.

The partially blinded dataset was subjected to the following screening in Microsoft Excel by CS prior to implementation of the analysis protocol by FM. CS coded responses to attention checks as correct or incorrect. Rows with both attention checks incorrect were to be removed from the dataset. There were no cases of both attention checks being incorrect.

Rows with one incorrect attention check (43 in total) and one or more incorrect or partially incorrect manipulation check (38 in total) were to be included in the dataset but flagged for a subsequent sensitivity analysis in which the same analysis procedure was carried out with the flagged rows removed. Rows that met these criteria were not excluded if the participant contacted the research team to explain that they had responded to the attention check incorrectly in error rather than due to lack of attention. This meant that the resulting number of rows to exclude for sensitivity analysis was 31.

Variable and level names were unblinded on completion of the primary analyses and initial significance interpretation. Analyses were repeated with the originally downloaded Qualtrics data to check that no error had been introduced by the blinding procedure. A proportion of reported analyses were also completed after unblinding. These included post hoc, exploratory manipulation checks and the exploratory tests of effect of conditions on SDT variables. This meant that the final significance interpretation with the Benjamini-Hochberg procedure was also completed post-unblinding.

For initial manipulation check analysis, responses to settings manipulation checks were recoded so that “Fully correct for Recreation condition” was entered for rows where all three recreation settings mentioned in the scenario were selected and no other settings were selected and “Fully correct for Health condition” was entered for rows where both health settings mentioned in the scenario were selected and no other settings were selected. “Partially correct” for either settings condition was entered where k-1 recreation or health settings mentioned in the scenario were selected and no other options selected. Responses to policy settings were subjected to recoding, whereby selecting options corresponding to all types of measures required for access to settings (and selecting no other options) constituted a fully correct response. For the condition in which an LFT would be required at cost, an additional requirement for coding as fully correct was an option other than “free” being selected. For the condition in which a free LFT would be required, a fully correct response could only be achieved if the option “free” was selected. “Partially correct” for any experimental condition meant that all the measures that were only cited in their specific condition had been selected (plus one or none of the only measures cited in the no-certification condition) and no dummy options selected. “Partially correct” for the no-certification condition meant that at least one of the measures cited in all conditions had been selected and no other options selected.

Chi-square tests indicated a significant relationship between setting condition and the frequency of fully or partially correct responses to the question around setting, and between the type of certification and countermeasures identified by participants, χ^2^(4) = 2292.62, p < .001, and between policy condition and frequency of fully or partially correct responses to policy manipulation check responses, χ^2^(24) = 3201.12, p < .001. However, on reviewing the breakdown of results, the research team agreed that this was not a clear indication as to whether participants had comprehended the implications of the scenario they had read. Therefore post hoc chi-squares were carried out post-unblinding. These are the analyses reported and described in the main manuscript. The two p values reported above were included, along with the p values of the reported manipulation checks, in the Benjamini-Hochberg calculation (see Supplementary File 5).
