# Supplementary material for: An online RCT on behavioural expectations effects of COVID-19 certification policies in England
Source: Vaccine X. 2023 Sep 20;15:100389. doi: 10.1016/j.jvacx.2023.100389 (PMC10565557; doi:10.1016/j.jvacx.2023.100389)
Supplement: Supplementary Data 5 [file mmc5.docx]

**Online RCT on Behavioural Expectations Effects of COVID-19 Certification Policies in England**

**Supplementary File 5 – Benjamini Hochberg Procedure**

**Table 2:**

*Output of the Benjamini Hochberg Procedure*

| **Test Description** | ***p*** | ***i*** | ***q*** | ***m*** | **crit** | **test** |
| --- | --- | --- | --- | --- | --- | --- |
| Confident ANCOVA Flu Vax SettingxPolicy | .990 | 65 | 0.1 | 65 | 0.1 | Not Significant |
| Hesitant ANCOVA C19 Vax Setting | .975 | 64 | 0.1 | 65 | 0.098461538 | Not Significant |
| ANCOVA C19 Vax Setting | .919 | 63 | 0.1 | 65 | 0.096923077 | Not Significant |
| Confident ANCOVA C19 Vax Policy | .885 | 62 | 0.1 | 65 | 0.095384615 | Not Significant |
| Hesitant ANCOVA Flu Vax Setting | .880 | 61 | 0.1 | 65 | 0.093846154 | Not Significant |
| Moderation Policy C19 Vax | .866 | 60 | 0.1 | 65 | 0.092307692 | Not Significant |
| Confident ANCOVA Flu Vax Policy | .865 | 59 | 0.1 | 65 | 0.090769231 | Not Significant |
| Moderation Policy Adherence | .853 | 58 | 0.1 | 65 | 0.089230769 | Not Significant |
| Moderation Setting Flu Vax w covariates | .853 | 57 | 0.1 | 65 | 0.087692308 | Not Significant |
| Confident ANCOVA C19 Vax SettingxPolicy | .847 | 56 | 0.1 | 65 | 0.086153846 | Not Significant |
| Moderation Setting C19 Vax w covariates | .843 | 55 | 0.1 | 65 | 0.084615385 | Not Significant |
| Moderation Policy Adherence w covariates | .832 | 54 | 0.1 | 65 | 0.083076923 | Not Significant |
| ANCOVA C19 Vax Policy | .829 | 53 | 0.1 | 65 | 0.081538462 | Not Significant |
| Hesitant ANCOVA Adherence Policy | .821 | 52 | 0.1 | 65 | 0.08 | Not Significant |
| Confident ANCOVA C19 Vax Setting | .803 | 51 | 0.1 | 65 | 0.078461538 | Not Significant |
| Moderation Policy C19 Vax w covariates | .800 | 50 | 0.1 | 65 | 0.076923077 | Not Significant |
| Hesitant ANCOVA C19 Vax Policy | .763 | 49 | 0.1 | 65 | 0.075384615 | Not Significant |
| Confident ANCOVA Adherence SettingxPolicy | .707 | 48 | 0.1 | 65 | 0.073846154 | Not Significant |
| Confident ANCOVA Adherence Setting | .697 | 47 | 0.1 | 65 | 0.072307692 | Not Significant |
| ANCOVA Adherence Policy | .670 | 46 | 0.1 | 65 | 0.070769231 | Not Significant |
| Visualisation 2: Policy | .641 | 45 | 0.1 | 65 | 0.069230769 | Not Significant |
| ANOVA Relatedness: Setting | .639 | 44 | 0.1 | 65 | 0.067692308 | Not Significant |
| Visualisation 1: Setting | .638 | 43 | 0.1 | 65 | 0.066153846 | Not Significant |
| Moderation Policy Flu Vax | .616 | 42 | 0.1 | 65 | 0.064615385 | Not Significant |
| ANOVA Competence: Setting | .606 | 41 | 0.1 | 65 | 0.063076923 | Not Significant |
| Confident ANCOVA Flu Vax Setting | .555 | 40 | 0.1 | 65 | 0.061538462 | Not Significant |
| Confident ANCOVA Adherence Policy | .545 | 39 | 0.1 | 65 | 0.06 | Not Significant |
| ANCOVA C19 Vax SettingxPolicy | .539 | 38 | 0.1 | 65 | 0.058461538 | Not Significant |
| Moderation Policy Flu Vax w covariates | .535 | 37 | 0.1 | 65 | 0.056923077 | Not Significant |
| ANCOVA Flu Vax Setting | .516 | 36 | 0.1 | 65 | 0.055384615 | Not Significant |
| Visualisation 2: Setting | .508 | 35 | 0.1 | 65 | 0.053846154 | Not Significant |
| Visualisation 1: Policy | .502 | 34 | 0.1 | 65 | 0.052307692 | Not Significant |
| Hesitant ANCOVA Adherence SettingxPolicy | .422 | 33 | 0.1 | 65 | 0.050769231 | Not Significant |
| ANCOVA Flu Vax SettingxPolicy | .373 | 32 | 0.1 | 65 | 0.049230769 | Not Significant |
| ANOVA Autonomy: PolicyxSetting | .257 | 31 | 0.1 | 65 | 0.047692308 | Not Significant |
| ANCOVA Adherence SettingxPolicy | .251 | 30 | 0.1 | 65 | 0.046153846 | Not Significant |
| ANCOVA Adherence Setting | .244 | 29 | 0.1 | 65 | 0.044615385 | Not Significant |
| Visualisation 2: PolicyxSetting | .234 | 28 | 0.1 | 65 | 0.043076923 | Not Significant |
| ANOVA Relatedness: Policy | .228 | 27 | 0.1 | 65 | 0.041538462 | Not Significant |
| ANOVA Relatedness: PolicyxSetting | .217 | 26 | 0.1 | 65 | 0.04 | Not Significant |
| ANOVA Autonomy: Setting | .213 | 25 | 0.1 | 65 | 0.038461538 | Not Significant |
| Moderation Autonomy | .204 | 24 | 0.1 | 65 | 0.036923077 | Not Significant |
| ANCOVA Flu Vax Policy | .191 | 23 | 0.1 | 65 | 0.035384615 | Not Significant |
| Moderation Setting C19 Vax | .188 | 22 | 0.1 | 65 | 0.033846154 | Not Significant |
| Hesitant ANCOVA Flu Vax Policy | .170 | 21 | 0.1 | 65 | 0.032307692 | Not Significant |
| Hesitant ANCOVA C19 Vax SettingxPolicy | .162 | 20 | 0.1 | 65 | 0.030769231 | Not Significant |
| Visualisation 1: PolicyxSetting | .150 | 19 | 0.1 | 65 | 0.029230769 | Not Significant |
| Hesitant ANCOVA Flu Vax SettingxPolicy | .131 | 18 | 0.1 | 65 | 0.027692308 | Not Significant |
| Moderation Setting Flu Vax | .114 | 17 | 0.1 | 65 | 0.026153846 | Not Significant |
| ANOVA Competence: Policy | .095 | 16 | 0.1 | 65 | 0.024615385 | Not Significant |
| Hesitant ANCOVA Adherence Setting | .070 | 15 | 0.1 | 65 | 0.023076923 | Not Significant |
| ANOVA Competence: PolicyxSetting | .039 | 14 | 0.1 | 65 | 0.021538462 | Not Significant |
| Moderation Setting Adherence | .035 | 13 | 0.1 | 65 | 0.02 | Not Significant |
| Moderation Setting Adherence w covariates | .021 | 12 | 0.1 | 65 | 0.018461538 | Not Significant |
| Per Protocol Manipulation Check 1 (Setting) | .000 | 11 | 0.1 | 65 | 0.016923077 | Significant |
| Per Protocol Manipulation Check 2 (Policy) | .000 | 10 | 0.1 | 65 | 0.015384615 | Significant |
| Exploratpry Manipulation Check 1 (vaccine) | .000 | 9 | 0.1 | 65 | 0.013846154 | Significant |
| Exploratpry Manipulation Check 2 (negative LFT) | .000 | 8 | 0.1 | 65 | 0.012307692 | Significant |
| Exploratpry Manipulation Check 3 (care homes) | .000 | 7 | 0.1 | 65 | 0.010769231 | Significant |
| Exploratory Manipulation Check 4 (hospitals) | .000 | 6 | 0.1 | 65 | 0.009230769 | Significant |
| Exploratory Manipulation Check 5 (nightclubs) | .000 | 5 | 0.1 | 65 | 0.007692308 | Significant |
| Exploratory Manipulation Check 6 (large indoor events) | .000 | 4 | 0.1 | 65 | 0.006153846 | Significant |
| Exploratory Manipulation Check 7 (large outdoor events) | .000 | 3 | 0.1 | 65 | 0.004615385 | Significant |
| Exploratory Manipulation Check 8 (expected costs of LFTs) | .000 | 2 | 0.1 | 65 | 0.003076923 | Significant |
| ANOVA Autonomy: Policy | .000 | 1 | 0.1 | 65 | 0.001538462 | Significant |

*Note: p = significance value from statistical analyses conducted on SPSS; i = the individual p-value’s rank; m = the total number of tests; Q = the false discovery rate; crit = critical value*
